# Barriers to agricultural employment among African American youth: Evidence from the American Community Survey

**DOI:** 10.1371/journal.pone.0330114

**Published:** 2025-08-22

**Authors:** Anthony Baffoe-Bonnie, Fafanyo Asiseh, Obed Quaicoe

**Affiliations:** 1 Department of Agricultural Economics, Texas A&M University, College Station, Texas, United States of America; 2 Department of Economics, North Carolina A&T State University, Greensboro, North Carolina, United States of America; 3 Department of Agribusiness, Applied Economics, and Agriscience Education, North Carolina A&T State University, Greensboro, North Carolina, United States of America; University of Professional Studies, GHANA

## Abstract

This paper investigates the occupational choices of African American youth in U.S. agricultural and food sectors. Using nationally representative data from the American Community Survey, we estimate a multinomial logit model to assess how socioeconomic conditions influence employment in three occupational categories: farming, farm-related work, and food preparation. Results reveal that agricultural employment among African American youth remains rare, and that gender is a strong predictor of occupational choice. Young African American women are significantly less likely than men to work in farming or related sectors, and more likely to be employed in food preparation. Educational attainment and student status are positively associated with food service employment but do not predict participation in farming occupations. These findings have important implications for agricultural policy, particularly as policymakers seek to address demographic disparities and revitalize the rural workforce. The results highlight the need for youth-specific policies, including targeted outreach, farm incubator programs, and access to capital, that address the compounded barriers facing youth in agricultural employment.

## 1 Introduction

The U.S. agricultural sector faces urgent labor challenges, including workforce shortages and difficulties attracting and retaining young workers. These challenges are amplified by a rapidly aging workforce—now averaging nearly 58 years—raising concerns about who will operate the farms of the future [1]. These demographic challenges are compounded by the lack of diversity across the farming population. According to the USDA’s 2017 Census of Agriculture, more than 64% of principal farm operators are male, and over 95% identify as White. The current composition of the agricultural workforce raises questions about how the next generation of agricultural leaders is being developed.

The challenge is especially pronounced for Black Americans, who have historically faced barriers to accessing agricultural land ownership, technical assistance, and farm financing [2, 3]. Although federal and state programs—such as the USDA’s 2501 Program and the Beginning Farmer and Rancher Development Program—have sought to increase participation of minority groups, young African Americans remain significantly underrepresented in both farming and food system employment. This persistent gap calls into question the reach and efficacy of existing outreach and workforce development efforts and highlight the need for a deeper understanding of the barriers to entry faced by the youth (for statistical purposes, youth are defined as persons between the ages of 15–24 years [4]).

The agriculture and food sectors offer a wide range of employment opportunities beyond traditional farm ownership, including roles in production, processing, and food service. Yet we know little about how young people—especially those from historically underserved communities—navigate these occupational pathways. African American youth face constraints that limit their access to agricultural careers. Cultural perceptions, social conditions, financial limitations, and lack of exposure to farming are widely cited as contributing factors [5–8], but few studies have empirically examined how these factors influence actual employment decisions. This paper addresses that gap by examining the determinants of occupational choice among African American youth in the U.S. food and agriculture sectors. While African American youth are the central focus, we include White youth as a benchmark to highlight whether standard predictors—such as gender, education, and income—operate similarly across groups and to identify any differences in occupational outcomes.

Using data from the American Community Survey (ACS) from 2009 to 2019 and a multinomial logit model, we analyze how individual and household characteristics shape the likelihood of employment in three occupational categories: (i) farming (including operators and managers), (ii) farm-related labor (e.g., equipment operators, animal caretakers), and (iii) food preparation and service. These categories span upstream and downstream segments of the agricultural supply chain and reflect diverse points of entry into the sector. This study contributes to the literature by providing nationally representative evidence on how individual and household-level factors shape youth sorting into food system employment. It also offers empirical insights to guide policies promoting long-term labor sustainability in agriculture.

The remainder of the paper proceeds as follows. Sect 2 reviews the literature on barriers to youth inclusion and career decision in the agricultural sector. Sect 3 describes the ACS data and presents summary statistics. Sect 4 outlines the multinomial logit model and empirical strategy. Sect 5 presents results and discusses their implications for agricultural labor policy. Sect 6 concludes with recommendations for future research.

## 2 Barriers to youth inclusion in agriculture

Globally, young people face substantial barriers to entering agriculture, including limited access to land, financial capital, and relevant training [9, 10]. These constraints are particularly pronounced for youth without family ties to farming. Even when land is available through inheritance or rental markets, startup financing remains a major obstacle. Farming is often perceived by lenders as high-risk, and young applicants frequently lack the collateral or credit history required for loans [11]. Moreover, limited technical expertise or business experience can prevent youth from developing viable farm plans that meet institutional lending standards. In the U.S., these challenges are compounded by demographic trends. The average age of U.S. farmers continues to rise, with individuals over 65 outnumbering those under 35 by more than six to one [12]. Despite concerns about generational succession, youth engagement in agriculture remains limited. Initiatives like the National Young Farmers Coalition offer mentorship and small grants [12], but these efforts do not fully address the structural forces that exclude young people from agricultural careers.

Even when material barriers such as land access or financing are addressed, social, cultural and institutional dynamics continue to shape participation in agriculture. Persistent structures of gender bias influence who is perceived as legitimate or competent, limiting the inclusion of certain groups in farming professions [13]. Historical discrimination—such as the documented exclusion of African American, Latinx, and Native American farmers from USDA programs—has produced racial disparities in land ownership and farm viability [14–16]. Gendered norms further constrain participation: Basche and Carter [17] find that agronomy students often rely on stereotypes in conservation planning, which reinforce patriarchal assumptions about women landowners. These findings suggest that expanding access to land or training alone is insufficient. Genuine inclusion requires transforming norms that determine who is seen as ‘fit’ to farm.

### 2.1 Youth career decision and agricultural sector

The transition from adolescence to early adulthood (ages 16–24) is a formative period for occupational decision-making. Career trajectories during this phase are shaped by both structured influences—such as formal education, internships, and mentoring—and unstructured forces, including family instability or local labor market disruptions [18, 19]. In agriculture, early exposure and access to land, capital, and institutional support often determine who enters the profession. Youth from farming families benefit from inherited experience and assets, while those from non-farm or historically marginalized backgrounds face steeper entry barriers.

Despite these constraints, youth bring unique comparative advantages to agriculture. They are more likely than older cohorts to adopt precision technologies, engage with digital platforms, and adapt to global disruptions such as climate variability and supply chain shocks [20]. For example, during the COVID-19 pandemic, youth-led enterprises shifted their operations quickly to online distribution and community-supported agriculture (CSA) models, demonstrating their potential to drive resilience and innovation in food systems [21]. Youth participation in agriculture also supports broader public benefits: rural employment, food system renewal, and intergenerational farm succession.

Recognizing this potential, U.S. federal and state programs have introduced youth-focused agricultural education, technical training, and credit access. Yet participation among African American youth remains low [22]. Many of these programs overlook how factors such as socioeconomic status interact to influence access to agricultural opportunities. While investments in areas like education and credit are valuable, they may be insufficient to address persistent challenges related to land access, networks, and trust in institutions. To expand youth participation, particularly among underrepresented populations, agricultural policy must move beyond broad inclusion strategies and develop mechanisms that address specific barriers to entry and engagement [23]. This includes revisiting eligibility criteria, broadening outreach efforts, and redesigning school-to-career pathways to better align with the diverse experiences and needs of today’s youth.

In short, agricultural labor policy must treat youth not only as future workers, but as potential agents of transformation in food systems. Doing so requires empirically grounded insights into how young people sort into or out of agricultural employment under existing constraints. This study contributes to ongoing policy discussions around youth employment by identifying individual and household-level determinants of occupational sorting into three key segments of the agricultural labor market: farming, farm-related occupations, and food preparation.

## 3 Data and descriptive statistics

We use data from the American Community Survey (ACS) spanning 2009–2019. The ACS is a nationally representative, continuous survey of the U.S. population that provides rich microdata on employment, education, income, and demographic characteristics. Unlike the Census of Agriculture, which focuses narrowly on farm operators, the ACS captures a broader spectrum of agricultural and food system employment—making it well-suited for analyzing early career patterns among youth across the food supply chain. We restrict the sample to individuals aged 16 to 24 who self-identify as either Black (African American) or White and who are either employed or enrolled in school. The lower age limit of 16 reflects the legal minimum age for formal employment in most states. After applying these criteria, our final sample consists of 212,256 observations, enabling robust subgroup comparisons.

Table 1 presents summary statistics for key categorical variables. The overall sample is gender-balanced, though some racial differences are apparent: 56.6% of Black youth in the sample are male, compared to 59.7% of White youth. Educational attainment is broadly similar across racial groups (with the exception of the small “no schooling” category), with most respondents having completed high school and approximately one-third reporting some post-secondary education. However, differences in student enrollment are more pronounced. While 69.7% of White youth are enrolled in school, only 57.6% of Black youth are—potentially reflecting differences in access to higher education or financial constraints, both of which may shape occupational outcomes.

**Table 1:** Descriptive statistics of categorical variables.

		White		Black		Total	
Variable	Category	No.	%	No.	%	No.	%
Gender	Male	98,513	49.8	8,252	56.6	106,765	50.3
	Female	99,153	50.2	6,338	43.4	105,491	49.7
Education	No School	507	0.3	96	0.7	603	0.3
	Elementary	82	0.0	4	0.0	86	0.0
	Middle School	1,608	0.8	75	0.5	1,683	0.8
	High School	143,296	72.5	10,519	72.1	153,815	72.5
	Tertiary	52,173	26.4	3,896	26.7	56,069	26.4
Student Status	Not Enrolled	59,964	30.3	6,192	42.4	66,156	31.2
	Enrolled	137,702	69.7	8,398	57.6	146,100	68.8
Occupation	Farmer	2,554	1.3	19	0.1	2,573	1.2
	Farming-Related	18,366	9.3	293	2.0	18,659	8.8
	Food Services	176,746	89.4	14,278	97.9	191,024	90.0
	**Observations**	197,666		14,590		212,256	

Occupational patterns also differ by race. Farming (defined here to include farm operators and managers) accounts for only 1.2% of total youth employment, and just 0.1% among Black respondents. Farm-related roles (farming-related roles include supervisory or technical positions linked to agricultural production but not involving direct farm management. For example, equipment operators, graders, animal caretakers, etc.) comprise 8.8% of the total sample but only 2.0% of Black youth. In contrast, food preparation and service employment (food preparation and service includes cooks, waitstaff, fast-food workers, and similar roles in hospitality and food retail) dominates among all youth: 90.0% overall, including 97.9% of Black youth and 89.4% of White youth. These descriptive patterns highlight the overwhelming concentration of young people in downstream, low-wage segments of the food system, rather than in production agriculture or technical agricultural careers.

Table 2 reports descriptive statistics for key continuous variables. The average age of respondents is approximately 19 years, with minimal variation between Black and White youth. Family size is also comparable across groups, averaging just under four members. However, notable differences emerge in household economic resources. The mean family income among Black youth is $74,883, compared to $93,123 for White youth. The overall average family income in the sample is $91,870. Income disparities may influence career trajectories by affecting individuals’ capacity to enter capital-intensive fields such as farming, where high start-up costs and financial risk present substantial barriers to entry.

**Table 2:** Descriptive statistics of continuous variables (young people).

	White		Black		Total	
Variable	Mean	SD	Mean	SD	Mean	SD
Age	19.26	2.36	19.87	2.32	19.31	2.37
Family Size	3.99	1.46	4.03	1.62	3.99	1.47
Family Income	93,123.31	51,901	74,882.76	47,698.54	91,869.5	51,828.85

## 4 Empirical strategy

We employ a multinomial logit (MNL) model within a random utility framework to examine how individual and household characteristics influence youth occupational choices in the agricultural sector. The dependent variable captures one of three mutually exclusive employment categories: (1) farming (e.g., operators and managers), (2) farm-related occupations (e.g., equipment operators, animal caretakers), and (3) food preparation and service, which serves as the base category. We use food preparation as the reference category for three reasons. First, it is the most prevalent form of employment among youth, particularly those facing barriers to higher-skilled or asset-intensive careers. Food preparation typically requires limited formal education and offers widespread availability, making it a realistic alternative for young workers. Second, using food service as the base enables policy-relevant comparisons between low-barrier and higher-barrier agricultural occupations. This allows us to assess the factors that enable (or constrain) transitions into more capital-intensive agricultural roles. Third, this allows us to focus the analysis on active labor market participants, offering a clearer behavioral comparison across occupational types. The MNL model is well-suited for this analysis because the outcome variable comprises unordered categorical responses. It allows us to estimate the relative likelihood of selecting each occupational type as a function of covariates while accounting for the absence of a natural ordering among categories. Although the model assumes the independence of irrelevant alternatives (IIA), its interpretability, alignment with the theoretical framework, and widespread application in occupational choice studies support its use here.

To explore whether occupational choice vary by race, we estimate separate models for African American and White youth. This stratified approach enables group-specific inference and avoids imposing uniform predictor effects across populations. While African American youth remain the central focus, the White youth subsample serves as a contextual benchmark to aid in interpreting observed differences across groups. We do not perform formal statistical comparisons across groups, but instead adopt a descriptive strategy to highlight differences in how key variables—such as gender, education, and income—are associated with occupational outcomes.

Each individual *i* in our sample is assumed to derive latent utility from choosing occupation *j*, which can be expressed as:

$$U_{ij}=X_{i}^{\prime }\beta_{j}+\varepsilon_{ij},$$ (1)

where *U*_*ij*_ denotes the latent utility individual *i* receives from choosing occupation *j*, *X*_*i*_ is a vector of observed individual- and household-level characteristics, $\beta_{j}$ is a vector of parameters to be estimated for category *j*, and $\varepsilon_{ij}$ is a stochastic error term. Under the assumption that $\varepsilon_{ij}$ is independently and identically distributed across alternatives, the probability that individual *i* chooses occupation *j* is given by:

$$\mathrm{Pr}(y_{i}=j|X_{i})=\frac{\exp(X_{i}^{\prime }\beta_{j})}{\sum_{m=1}^{J}\exp(X_{i}^{\prime }\beta_{m})},$$ (2)

where *J* = 3 represents the total number of occupational categories. To ensure model identification, we normalize $\beta_{3}=0$, corresponding to the base category (food preparation). Therefore, the estimated probabilities simplify to:

$$\mathrm{Pr}(y_{i}=j|X_{i})=\frac{\exp(X_{i}^{\prime }\beta_{j})}{1+\sum_{k=1}^{2}\exp(X_{i}^{\prime }\beta_{k})},\;\text{for }j=1,2,$$ (3)

and the probability of choosing the base category is:

$$\mathrm{Pr}(y_{i}=3|X_{i})=\frac{1}{1+\sum_{k=1}^{2}\exp(X_{i}^{\prime }\beta_{k})}.$$ (4)

Model parameters are estimated via maximum likelihood. The estimation is done using standard statistical software, with robust standard errors reported to address potential heteroskedasticity. The estimated coefficients from the multinomial logit model are interpreted relative to the base outcome. A coefficient $\beta_{jk}$ indicates the change in the log–odds of choosing occupation *j* (either Farmer or Farming-related) versus food preparation, associated with a one-unit increase in covariate *X*_*k*_.

## 5 Results and discussion

Table 3 presents the estimated coefficients from the multinomial logit models for African American and White youth. These coefficients reflect how individual and household characteristics are associated with the likelihood of selecting either farming or farm-related occupations, relative to the base category of food preparation. As the model is nonlinear, coefficients represent changes in log-odds and are not directly interpretable as marginal effects. To enhance interpretability, we report average marginal effects in Table 4.

**Table 3:** Multinomial model estimates of occupational choice.

	White Americans		African-Americans	
	Farming	Farming-Related	Farming	Farming-Related
	1	2	1	2
Age	0.197^***^	–0.104^***^	0.0201	–0.0482
	(0.0159)	(0.0122)	(0.0800)	(0.0569)
Gender (Female)	–1.756^***^	–1.380^***^	–2.446^**^	–1.037^***^
	(0.115)	(0.0537)	(0.753)	(0.224)
Student	–0.970^***^	–0.426^***^	–0.248	–0.425^*^
	(0.0852)	(0.0625)	(0.406)	(0.194)
Family size	0.00832	0.101^***^	–0.0844	0.0640
	(0.0443)	(0.0201)	(0.165)	(0.0652)
Family income	–0.0664	–0.203^***^	–0.206	–0.377^**^
	(0.0599)	(0.0383)	(0.261)	(0.144)
Education	0.0227	–0.240^***^	–0.00899	–0.208
	(0.112)	(0.0485)	(0.338)	(0.141)
Constant	–6.837^***^	3.171^***^	–3.394	1.997
	(0.782)	(0.552)	(4.556)	(1.428)
*N*	197,666		14,590	

**Table 4:** Marginal effects.

	Farming		Farming-Related		Food Prep./Service	
	(1)	(2)	(1)	(2)	(1)	(2)
	White	African	White	African	White	African
	Americans	Americans	Americans	Americans	Americans	Americans
Age	0.00206^***^	0.0000284	–0.00699^***^	–0.000851	0.00493^***^	0.000823
	(0.000315)	(0.000108)	(0.00107)	(0.000994)	(0.000966)	(0.000978)
Female	–0.0137^***^	–0.00197^**^	–0.0818^***^	–0.0158^***^	0.0955^***^	0.0177^***^
	(0.00169)	(0.000653)	(0.00703)	(0.00242)	(0.00832)	(0.00237)
Student	–0.00900^***^	–0.000315	–0.0285^***^	–0.00751^*^	0.0375^***^	0.00782^*^
	(0.00118)	(0.000545)	(0.00475)	(0.00337)	(0.00504)	(0.00326)
Family size	–0.0000177	–0.000115	0.00656^***^	0.00113	–0.00655^***^	–0.00102
	(0.000425)	(0.000226)	(0.00134)	(0.00120)	(0.00164)	(0.00116)
Family income	–0.000458	–0.000262	–0.0132^***^	–0.00665^*^	0.0136^***^	0.00691^*^
	(0.000563)	(0.000361)	(0.00284)	(0.00280)	(0.00321)	(0.00281)
Education	0.000465	–0.00000521	–0.0157^***^	–0.00367	0.0152^***^	0.00368
	(0.00109)	(0.000452)	(0.00306)	(0.00253)	(0.00376)	(0.00255)
*N*	197,666	14,590	197,666	14,590	197,666	14,590

Standard errors in parentheses.  *p*<0.05, ^**^ *p*<0.01, ^***^ *p*<0.001.

Among African American youth, relatively few predictors are statistically significant. Age is not statistically significant in predicting occupational choice for African American youth, possibly due to the limited variation in age within the sample. In contrast, gender shows a strong and statistically significant association: African American females are substantially less likely than their male counterparts to be employed in farming or farm-related roles, relative to food preparation. This persistent gender gap reflects not only differences in access to financial support and federal assistance programs [24], but also social norms that may disadvantage women in agricultural settings. Prior studies have shown that U.S. agriculture remains highly gendered, with farm inheritance and ownership frequently passed down to sons over daughters [15, 16].

Student status and educational attainment are not significantly associated with farming participation for African American youth, suggesting that conventional human capital indicators may have limited explanatory power in this context. These null results may indicate underlying constraints or suggest that the returns to education in agricultural pathways are limited for this subgroup. Household-level variables show mixed effects: family size is not significantly related to occupational choice, but family income is negatively associated with farm-related employment and positively associated with food preparation work. This may indicate that higher-income households are less reliant on agricultural jobs or that youth from such households face a different set of labor market opportunities.

For White youth, more predictors are statistically significant. Age is positively associated with farming participation but negatively associated with farm-related employment. Gender effects persist, with White females less likely to engage in farming or farm-related work and more likely to work in food preparation. Student status is negatively associated with both farming and farm-related occupations, suggesting that youth enrolled in school gravitate toward food preparation roles, possibly due to their flexibility with academic schedules. Educational attainment is negatively associated with farm-related work.

The negative association between student status and farming likely reflects broader cultural and class-based dynamics. For many youth, especially those pursuing higher education, farming is not perceived as a forward-looking or desirable career path. Education is often seen as a vehicle for exiting rural life and pursuing professional careers in urban areas. Among higher-income or landowning families, farmland may be retained as a financial asset, but it is not necessarily expected that children will become the primary operators. In such cases, families may rent out their land or rely on hired labor. These patterns suggest that farming remains culturally undervalued as a legitimate career for educated youth, helping to explain why student status is not positively associated with agricultural participation for either group.

Table 4 presents average marginal effects to facilitate interpretation. For African American youth, age and education remain insignificant. Gender shows a clear effect: being female reduces the probability of farming by 0.2 percentage points and farm-related work by 1.6 percentage points, while increasing the probability of food preparation employment by 1.8 percentage points. Family income also shows significant effects—negatively associated with farm-related employment and positively with food preparation—highlighting income-based sorting mechanisms.

For White youth, marginal effects are more consistent. Age increases the probability of farming and decreases the likelihood of farm-related roles. Female youth are significantly less likely to participate in farming or farm-related occupations and more likely to work in food preparation.

While targeted programs for women in agriculture have grown in recent years, our findings show that these initiatives can be complemented with changes that address barriers related to sectoral recognition. Studies have documented how women in agriculture continue to face lower pay, limited access to credit and advisory services, and exclusion from farm succession [15, 16]. In the absence of broader reforms, programs designed to support women may unintentionally place them in environments where discriminatory practices continue.

In summary, most individual and household characteristics predict occupational choice among White youth, however, their predictive power is more limited for African American youth. This limited predictive power may reflect constraints—such as lack of access to land, capital, or institutional networks—that are not directly captured in the ACS data but have been identified in prior research as key barriers to agricultural participation among marginalized groups [23]. Gender and family income emerge as the most consistent predictors across both groups. Education and student status, however, play more limited roles—particularly for African American youth. These findings highlight the need for agricultural workforce policies that go beyond informational or educational interventions to address the constraints in the broader food system.

## 6 Conclusion

This study investigates the occupational choices of youth in the U.S. agricultural and food sectors, with a particular focus on African American youth. Using nationally representative data from the American Community Survey (2009–2019) and a multinomial logit model, we analyze how individual and household characteristics—such as gender, education, family income, and student status—influence the likelihood of employment in three occupational categories: farming, farm-related work, and food preparation. By estimating separate models for African American and White youth, we highlight key patterns while maintaining focus on the barriers affecting African American youth.

Our findings reveal that agricultural employment is rare among youth overall and exceptionally uncommon among African Americans. Gender consistently predicts occupational sorting, with young women significantly less likely than men to enter farming or farm-related roles and more likely to work in food preparation. For African American youth, education and student status are not strong predictors of agricultural participation, and family income is negatively associated with the likelihood of farm-related employment. These results suggest that conventional workforce development strategies, such as improving access to education, are unlikely to be sufficient on their own to address exclusion from upstream agricultural careers.

The findings highlight the importance of responsive outreach, the development of school-to-career pipelines, and community-based agricultural programs that engage the youth on their own terms. Therefore, agricultural stakeholders should reconsider the implementation of training and support initiatives to ensure they effectively address the needs of young participants. These supports must go beyond increasing awareness or expanding training opportunities; they must directly confront the institutional and structural barriers that limit access for this groups. Future research could explore additional explanatory factors, including local labor market dynamics, land access, credit constraints, and participation in federal and state programs, to further explain the mechanisms of occupational sorting and identify more effective interventions. Ultimately, ensuring access to meaningful opportunities in agriculture requires more than interventions. It also demands a rethinking of how agricultural careers are defined, supported, and valued, particularly for those excluded from the sector.
